# An chromosome-level haplotype-resolved genome assembly and annotation of pitaya (*Selenicereus polyrhizus*)

**DOI:** 10.1038/s41597-025-04678-6

**Published:** 2025-04-01

**Authors:** Juncheng Li, Wenlong Luo, Biao Jiang, Satish Kumar, Mengfei Lin, Qingming Sun

**Affiliations:** 1 Institute of Fruit Tree Research, Guangdong Academy of Agricultural Sciences; Key Laboratory of South Subtropical Fruit Biology and Genetic Resource Utilization, Ministry of Agriculture and Rural Affairs; Guangdong Provincial Key Laboratory of Science and Technology Research on Fruit Tree, Guangzhou, 510640 China; 2 Guangdong Key Laboratory for New Technology Research of Vegetables, Vegetable Research Institute, Guangdong Academy of Agricultural Sciences, Guangzhou, 510640 China; 3 The New Zealand Institute for Plant & Food Research Limited, Private Bag 1401, Havelock North, 4157 New Zealand; 4 Jiangxi Provincial Key Laboratory of Plantation and High Valued Utilization of Specialty Fruit Tree and Tea, Institute of Biological Resources, Jiangxi Academy of Sciences, Nanchang, China

**Keywords:** Plant breeding, Plant genetics

## Abstract

Pitaya, (*Selenicereus spp*.), a fruit originating from North and Central America and extensively cultivated in China and Vietnam, holds significant economic value. Utilizing PacBio HiFi sequencing and Oxford Nanopore Technologies ultra-long sequencing, aided by Hi-C data, we have assembled a chromosome-level haplotype-resolved genome. The sizes of the two haplotype genomes were determined to be 1.477 Gb (hap1, contig N50 = 133.35 Mb) and 1.442 Gb (hap2, contig N50 = 132.57 Mb), with 96.7% (hap1) and 98.4% (hap2) respectively allocated to 11 pseudochromosomes. Hap1 comprises 58.94% repeat sequences and predicts a total of 29,139 protein-coding gene models and 18,378 non-coding RNAs. Hap2 comprises 58.37% repeat sequences and predicts a total of 28,538 protein-coding gene models and 19,458 non-coding RNAs. Notably, 93.5% and 93.6% of protein-coding genes were annotated for the two haplotypes. The high-quality genome assembly presented in this study provides a valuable resource for future ecological, evolutionary, biological, and breeding research in pitaya.

## Background & Summary

Pitaya, also known as “pitahaya” or “dragon fruit”, refers to the fruits of several species in the genus *Selenicereus* of the family Cactaceae. It is a rapidly growing and economically promising emerging fruit. Although there is no definitive evidence, it is widely believed that pitaya originated in North America and has been a traditional fruit in that region^1^. In recent years, pitaya has gained popularity in Asian countries, especially in Vietnam and China, where it has become a major fruit crop. In 2021, China surpassed Vietnam in terms of cultivation area, becoming the country with the largest pitaya cultivation area^2^. Pitaya is not only a delicious fruit but also rich in nutrients such as vitamin C, fiber, and various minerals. However, our understanding of the biological background of pitaya is still limited. Genome sequencing has played a crucial role in advancing various aspects of basic biology, and a high-quality reference genome can greatly enhance our understanding of the genetic basis and evolutionary processes underlying the biological characteristics of pitaya. While two versions of the pitaya genome, with genome sizes of 1.41 Gb and 1.33 Gb, N50 values of 127.15 Mb and 109.7 Mb, have recently been published^3,4^, further enhancements are required in terms of genome assembly completeness and annotation. In-depth studies on the pitaya genome will help uncover its genetic characteristics, interspecific differences, and gene regulatory networks, thus promoting genetic improvement and commercial cultivation of pitaya.

The aim of this study was to sequence, assemble, and annotate the genome of pitaya to obtain comprehensive genomic information and explore its genetic diversity and biological characteristics, providing strong support for genetic improvement and conservation of pitaya. Using PacBio long reads, nanopore ultra-long reads, and high-throughput chromosome conformation capture (Hi-C) data, we assembled the data using the hifiasm2 software^5^, resulting in a chromosome-level high-quality monoploid resolution genome assembly for pitaya. The two haplotypes had sizes of approximately 1.47 Gb and 1.44 Gb, respectively, and included 21 telomeres, nearly reaching the T2T level, with N50 values of 133.4 Mb and 132.6 Mb, respectively. Subsequent gene prediction and annotation identified a total of 34,673 genes, covering most regions of the pitaya genome. Through functional annotation and analysis of the pitaya genome, we discovered numerous genes related to its biological characteristics and agronomic traits, including fruit color, texture, quality, and disease resistance.

In addition, the genome assembly demonstrated a high level of completeness, with completeness scores of 97.7% and 94.4% for hap1 and hap2 genome, respectively, as determined by BUSCO analysis. Repetitive sequences accounted for 58% of the genome assembly. A total of 29,139 protein-coding genes were identified, with functional annotations available for 27,117 genes. Transcriptome analysis revealed that at least 24,893 genes were expressed in at least one tissue.

This study successfully sequenced, assembled, and annotated the genome of pitaya, providing an important foundation for further research on its genetic characteristics, biological features, and cultivation improvement. The decoding of the pitaya genome helps reveal its genetic characteristics and interspecific differences, thereby providing strong support for breeding improvement, variety identification, and resource conservation of pitaya, and promoting the sustainable development of the pitaya industry.

## Methods

### Sample collection and preparation

‘Dahong’ (*Selenicereus polyrhizus*) is one of the main cultivated varieties in China known for its excellent quality and high yield. The sequencing material selected for this study was a five-year-old pitaya variety ‘Dahong’ (Selenicereus polyrhizus) obtained from the germplasm nursery of the Fruit Research Institute, Guangdong Academy of Agricultural Sciences (113.3708° E, 23.1502° N) was chosen as the sequencing material. Multiple tissues including stem, tender shoots, flower bud, calyx, petals, style, stigma, filament, anther, pollen, fruit peel, fruit flesh, and root were collected from the same individual plant. These 13 samples were immediately frozen using liquid nitrogen and stored at −80 °C. Genomic DNA extraction and sequencing, as well as RNA sequencing, were carried out by Biomarker (Biomarker Technologies Co., LTD in Beijing, China).

### Genome and transcriptome sequencing

The genomic DNA for PacBio HiFi sequencing was extracted using the QIAamp DNA Mini Kit (QIAGEN) and processed according to the standard protocol provided by PacBio, which included sample quality assessment, library preparation, library quality assessment, and sequencing. In this project, the average CCS length exceeded 20,038 bp, with the longest CCS read achieving 56,993 bp. A total of 81.62 Gb (~56×) of valid data was obtained.

**Fig. 1:**
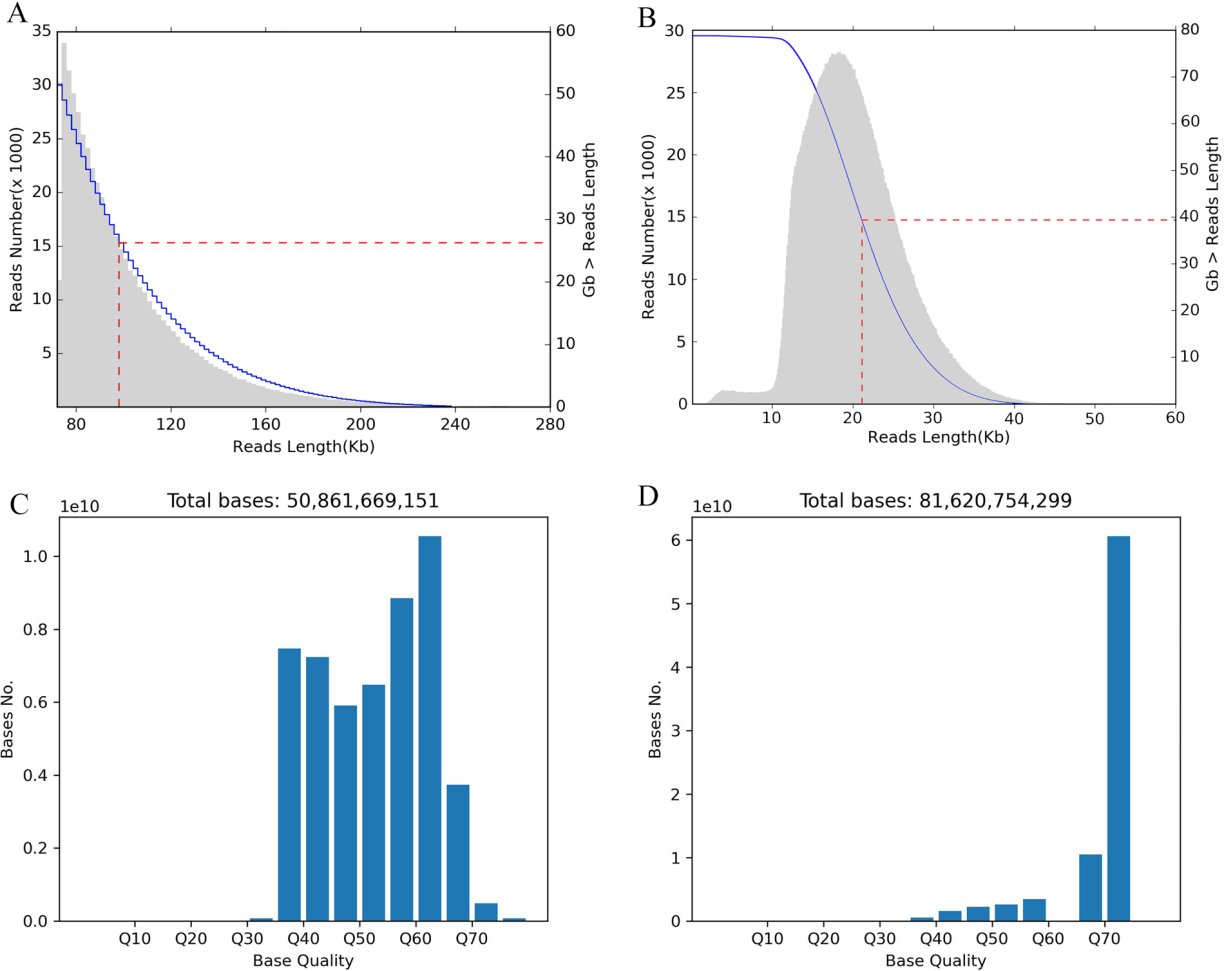
Read length and base quality distribution of the ONT ultra-long (**A,C**) and CCS Pacbio Hifi (**B,D**) sequencing data.

The Monarch® HMW DNA Extraction Kit for Tissue (New England Biolabs, T3060) was employed to extract high-quality DNA for Ultra-Long DNA Sequencing following the Kit protocol. The DNA library was constructed using the SQK-LSK109 Kit (Oxford Nanopore Technologies, Oxford, UK), followed by single-molecule sequencing of the DNA using GridION X5/PromethION. All procedures were conducted in accordance with the instruction manual. The effective ONT data output for this project was 52.65 Gb (~36×) with an average reads length 101,938 bp and the longest read achieving 907,782 bp. The statistical results are summarized in Table 1, the detail of reads length distribution can be found in Fig. 1.

**Table 1:** Length distribution of ONT ultra-long and CCS Pacbio Hifi reads.

Data Type	Reads number	Reads base	N50	Mean length	Max length
ONT Ultra-long	516,534	52,654,430,685	100,000	101,938	907,782
Pacbio Hifi	4,068,089	81,620,754,299	21,116	20,038	56,993

### Hi-C library preparation, sequencing and genome assembly

The Hi-C method is a chromosome conformation capture technique that integrates high-throughput sequencing^6^. The Hi-C library was prepared following the steps described below: DNA cross-linking using formaldehyde as a fixative agent, restriction enzyme cleavage using DpnII, introduction of biotin-labeled bases for end-repair, circularization, and DNA capturing and purification. The concentration and insert size of the library were examined using Qubit 2.0 and Agilent 2100, respectively. Q-PCR was further processed for accurate DNA quantification to ensure an adequate amount. For this Hi-C library, the ratio of truncated reads in total reads is about 30%, which is significantly higher than the general standard of not less than 10%, indicating a high-quality library. After sequencing, a total of 144.64 Gb (~100×) of Clean Data was obtained, with a Q30 ratio exceeding 93.70%.

### RNA library construction and transcriptome sequencing

Equal amounts of total RNA from all 13 samples, including stem, tender shoots, flower bud, calyx, petals, style, stigma, filament, anther, pollen, fruit peel, fruit flesh, and root, were pooled and sent to Biomarker Technologies Co., LTD in Beijing, China for library construction and sequencing. Two different sequencing platforms were employed, with Novaseq. 6000 Platform (150 bp paired-end mode), resulting in a total of 41.08 M reads and 12.29 Gb of clean data, with a Q30 base percentage of 94.05%. Full-length mRNA sequencing was conducted using the Oxford Nanopore Sequencing Platform, yielding 12.87 Gb of clean data, comprising a total of 10,729,994 sequences, with an N50 value of 1,423 bp.

### Chromosome-level genome assembly

The primary assembly utilized hifiasm v0.19.6-r595 software^5^ to integrate PacBio HiFi reads, Oxford Nanopore Technology ultra-long reads, and Hi-C reads, resulting in two distinct haplotype-resolved genome primary assemblies. Hi-C reads were aligned to the genomes of the two haplotypes using chromap^7^ v0.2.5-r473and samtools^8^ v1.20, followed by contig anchoring to chromosomes using yahs^9^ v1.2a.1 with Hi-C data. Manual adjustment of anchored results, refining chromosome segment boundaries, and correcting visible assembly errors was performed using Juicebox^10^ v1.11.08. Finally, nextpolish2^11^ v0.2.0 was employed with Hifi reads to fill gaps between contigs, yielding two chromosome-level, haplotype-resolved genome assemblies.

**Fig. 2:**
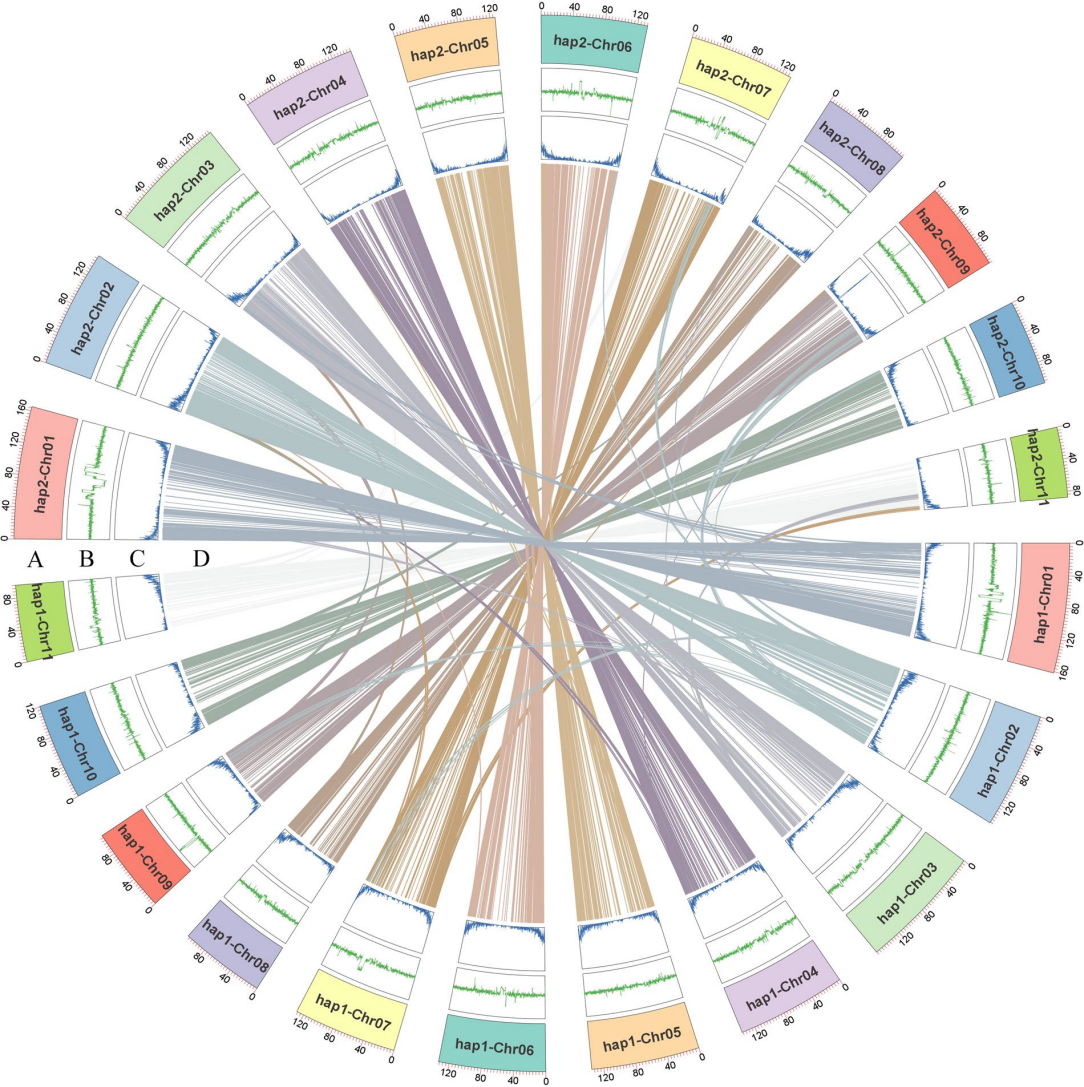
An overview of haplotype-resolved genome assembly of *Selenicereus polyrhizus*. (**A**) Chromosome ID and size, (**B**) GC content, (**C**) protein-coding gene density, and (**D**) analysis of collinearity.

Haplotype 1 consists of 11 chromosomes and 519 unanchored scaffolds, with an N50 value of 133.4 Mb and a genome size of 1.48 Gb. The 11 chromosomes range in size from 96.03 Mb to 164.45 Mb, collectively representing 96.7% of the total genome size (Fig. 2, Tables 2, 3).

**Table 2:** Assembly statistics for the two haplotypes.

Haplotype	Haplotype 1	Haplotype 2
Total_Len (bp)	1,477,356,928	1,441,923,378
Total_Seq_Num	530	211
Total_N_Counts	0	0
Total_LowCase_Counts	0	0
Total_GC_content	37%	37%
Minimum Len (bp)	1,000	1,000
Maximum Len (bp)	161,447,098	165,418,061
Mean Len (bp)	2,787,465.9	6,833,760.09
Median Len (bp)	73,375	88,566
N50 (bp)	133,352,528	132,574,350

**Table 3:** Chromosomes and 相应的 centromeres statistics for the two haplotypes.

Haplotype	Chr ID	Chr size	CM start	CM end	CM size
Haplotype 1	Chr01	1.61E + 08	115351853	115812900	461048
	Chr02	1.45E + 08	63374861	63498318	123458
	Chr03	1.57E + 08	130553817	132686269	2132453
	Chr04	1.37E + 08	66569492	71955425	5385934
	Chr05	1.35E + 08	55541143	57592768	2051626
	Chr06	1.33E + 08	60621477	64952033	4330557
	Chr07	1.29E + 08	52311659	52968551	656893
	Chr08	1.07E + 08	44554491	44718881	164391
	Chr09	1.07E + 08	29290175	31636530	2346356
	Chr10	1.21E + 08	29125168	29571904	446737
	Chr11	96031892	21522316	22098734	576419
Haplotype 2	Chr01	1.65E + 08	78252243	88114936	9862694
	Chr02	1.48E + 08	30742388	31385732	643345
	Chr03	1.49E + 08	58225049	59391393	1166345
	Chr04	1.45E + 08	64601860	68344250	3742391
	Chr05	1.27E + 08	87187917	87340381	152465
	Chr06	1.33E + 08	59908171	62885719	2977549
	Chr07	1.32E + 08	67626162	69764254	2138093
	Chr08	1.13E + 08	65716978	70379660	4662683
	Chr09	1.12E + 08	78756334	78892745	136412
	Chr10	1.07E + 08	66601714	67519072	917359
	Chr11	87842756	59474480	60337628	863149

Note: Note: In the table, “Chr” represents chromosome, and “CM” represents centromere.

Haplotype 2 comprises 11 chromosomes and 200 unanchored scaffolds, with an N50 value of 133.4 Mb and a genome size of 1.44 Gb. The 11 chromosomes range in size from 87.84 Mb to 165.42 Mb, collectively representing 98.4% of the total genome size (Fig. 2, Tables 2, 3).

**Fig. 3:**
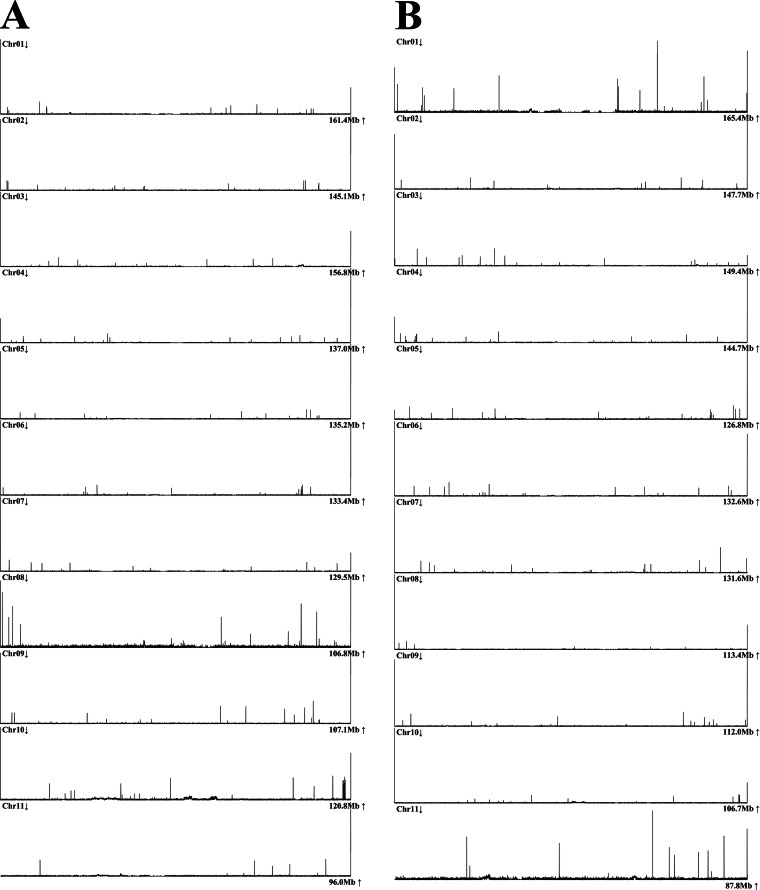
Telomere distribution in the assembly of two haploid genomes (Haplotype 1; Panel A, Haplotype 2; Panel B).

The CentroMiner and TeloExplorer function in quarTeT^12^ v1.2.1 with the “-c plant” option was utilized to identify telomeres and centromeres in the genome, detecting a total of 22 centromeres ranging from 123.5 kb to 9.86 Mb, and 42 telomeres with the sequence (AAACCCT) repeating at least 170 times in 10 kb regions at the ends of chromosomes, across 22 chromosomes in the two distinct haplotypes (Tables 3, 4). Interestingly, both haplotypes were found to have one telomere missing from the 11th chromosome (Fig. 3). In summary, this assembly can be regarded as having nearly achieved telomere-to-telomere and gap-free completeness.

**Table 4:** Repeat_number of telomeres sequence unit for the two haplotypes chromosomes.

Chromosome	Haplotype 1		Haplotype 2	
Chr01	2378	486	268	300
Chr02	1636	2018	940	2014
Chr03	2607	1010	1955	246
Chr04	429	1862	417	1786
Chr05	1277	2030	115	892
Chr06	2273	1560	1115	1530
Chr07	2357	351	1044	199
Chr08	224	265	1738	444
Chr09	746	1279	1143	1923
Chr10	527	348	1239	375
Chr11	—	989	—	170

### Identification of repetitive elements

To identify repeat sequences within the genome, a combined approach of de novo prediction and homology-based prediction was employed. For de novo prediction, long terminal repeat retrotransposons were first detected using LTRharvest^13^ (part of genometools^14^ v1.6.5) and LTRfinder^15^ to predict LTRs, and LTR_retriever^16^ v2.9.8 was used to filter and integrate the LTR predictions. MITE-Hunter^17^ was then utilized to predict miniature inverted-repeat transposable elements (MITEs), followed by a subsequent de novo prediction using RepeatModeler^18^ v2.0.4. The results from these steps were combined to produce the final de novo prediction. For homology-based prediction, RepeatMasker^19^ v4.1.6 and Tandem Repeats Finder^20^ (TRF v4.09) software were utilized. The de novo prediction and homology-based prediction results were then merged, and the bedtools^21^ v2.31.0 software was used to generate a soft-masked genome. Following the identification process, haplotype 1 was found to contain 58.94% repeat sequences, while haplotype 2 was found to contain 58.37% repeat sequences. This indicates that approximately 58% of the ‘Dahong’ genome sequence comprises repeat sequences.

### Gene identification and functional annotation

Gene identification is a complex and intricate process, and to ensure the accuracy and completeness of gene prediction and annotation, three strategies were utilized. Firstly, *ab initio* prediction was conducted using the SNAP^22^ (Semi-HMM-based Nucleic Acid Parser, version 2013-11-29) and glimmerHMM^23^ v3.0.4 software. Subsequently, homology-based prediction was performed using GeMoMa^24^, with the homologous protein library sourced from the model plants *Arabidopsis thaliana*, rice, and *Populus trichocarpa*. Furthermore, prediction based on direct evidence from RNA-seq was carried out, involving assembly of the RNA-seq data using Trinity^25^ and StringTie^26^, followed by integration of the assembly results using PASA^27^. The RNA-seq data was aligned to the genome using hisat2^28^ v2.31.0, and gene prediction was conducted based on the alignment results using the BRAKER3^29^ pipeline, which integrates GeneMark-EFP^30^, AUGUSTUS^31^ and TSEBRA^32^. Finally, the results obtained from the three different methods were integrated using EVidenceModeler^27^ v2.1.0 (EVM) to obtain the final prediction. Hap1 genome predicted 29,139 coding genes, while hap2 genome predicted 28,538 coding genes. The completeness of the prediction results was evaluated using BUSCO^33^ (V5.3.2) with the embryophyta_odb10 dataset, which indicated that the completeness of protein sequences for the two haplotypes reached 97.6% and 97.5%.

For functional annotation, we integrated the results from three independent methods. Firstly, the protein sequences were locally annotated for functional analysis using InterProScan^34^ v5.60 with the InterPro database (v100.0). Subsequently, BLAST comparisons were performed against UniProtKB/Swiss-Prot, UniProtKB/TrEMBL, NCBI nonredundant protein (NR), and *Arabidopsis databases* with an e-value threshold of 1e-5. Finally, gene sequences were mapped to the eggNOG^35^ v6.0 database using eggNOG-mapper^36^ v2.1.14 for protein annotation based on gene ontology (GO) terms. Upon statistical analysis of the annotation results, it was observed that 27,256 protein-coding genes (93.5%) in haplotype 1 and 26,703 protein-coding genes (93.6%) in haplotype 2 were annotated by at least one of the databases.

For non-coding RNA genes, we employed additional strategies for annotation and identification. Initially, Infernal^37^ v1.074 was used in conjunction with the Rfam database^38^ to retrieve non-coding RNA genes. Subsequently, tRNAscan-SE^39^ v2.0.972 and barrnap^40^ v0.973 (https://github.com/tseemann/barrnap) were employed to annotate tRNA and rRNA, respectively, using default parameters. Finally, the annotation results were merged, and redundant annotations were removed. In haplotype 1, we annotated 18,378 non-coding genes (11,723 rRNA, 5,215 tRNA, 101 miRNA, 396 snoRNA, and 943 others). In haplotype 2, we annotated 19,458 non-coding genes (14,948 rRNA, 2,870 tRNA, 102 miRNA, 684 snoRNA, and 854 others), the details can be found in Table 5.

**Table 5:** Protein coding Gene and Non-coding RNA annotation.

Categories	hap1	hap2
mRNA	29,139	28,538
Exon	158,431	157,337
Intron	129,292	128,810
Mean number of exons per mRNA	5.44	5.52
tRNA	5215	2870
rRNA	11723	14948
miRNA	101	102
snoRNA	396	684
Others	943	854

### Synteny analysis between haplotype assemblies

**Fig. 4:**
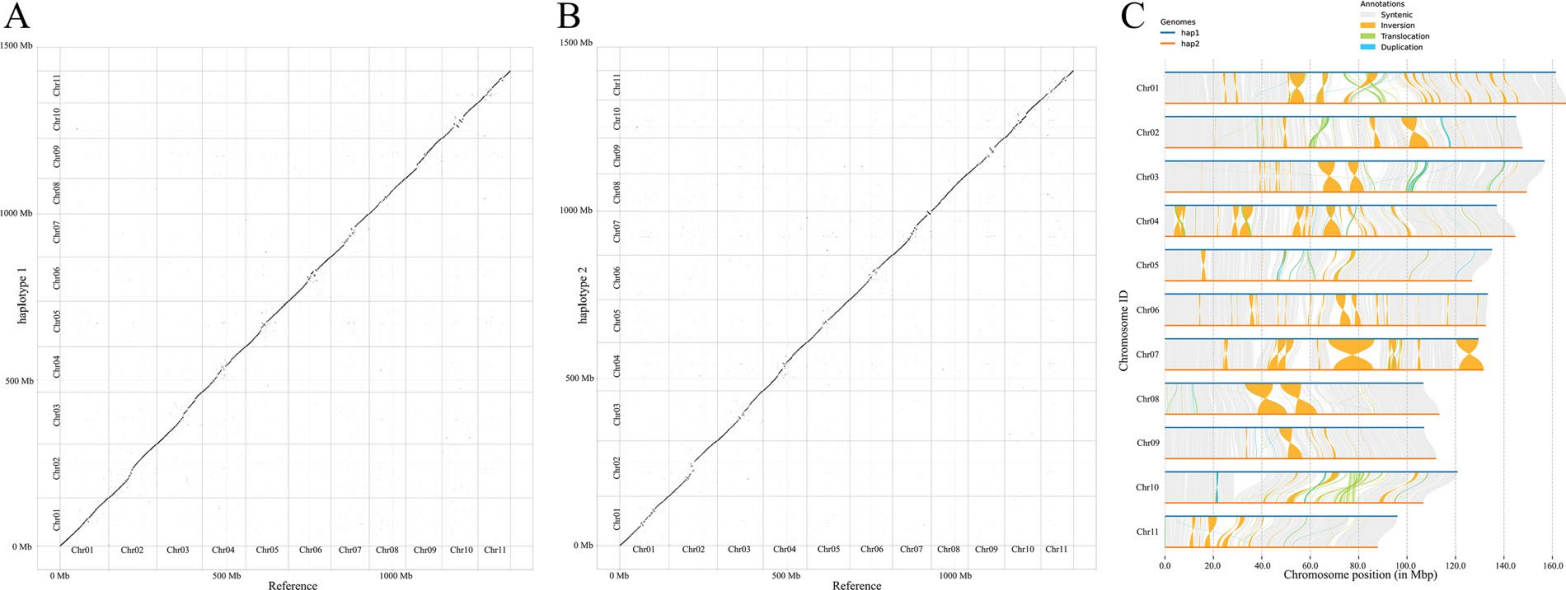
Comparison of two haplotypes with the reference genome. (**A**) Dot-plot of synteny blocks between Haplotype 1 and the reference genome; (**B**) Dot-plot of synteny blocks between Haplotype 2 and the reference genome; (**C**) Structural variations between Haplotype 1 and Haplotype 2.

The two haplotypes and a previously published chromosome-level genome^3^ (herein referred to as the reference genome) were aligned using minimap2^41^ with the parameter “-ax asm5”, and a dot plot illustrating the collinearity was generated using the R package ‘pafr’, highlighting the high similarity between the two haplotypes and the reference genome (Fig. 4A,B). Subsequently, the haplotypes were compared again using minimap2 with the parameters “-ax asm5–eqx”, and SyRI^42^ (Synteny and Rearrangement Identifier) v1.6.3 was employed to detect synteny and structural variations between the haplotypes (default parameters). The results were visualized using Plotsr^43^ v1.1.1, revealing a total of 1,946 syntenic regions (approximately 930 Mb), 214 inversions (approximately 173 Mb), 2,810 translocations (approximately 25.5 Mb), with 606 duplications (5.5 Mb) identified on haplotype 1 and 2,237 duplications (9.7 Mb) detected on haplotype 2 (Fig. 4C, Table 6). Additionally, 9,487,343 SNPs, 337,977 insertions, and 347,207 deletions were identified (Table 6). We speculate that the observed genetic variations between the two haplotypes may be attributed to potential interspecific hybridization among several *Selenicereus* species. Furthermore, we cannot dismiss the possibility that ‘Dahong’ is a product of multi-species hybridization, which could serve as a source of genetic variation between the two haploid genomes.

**Table 6:** Details of structural and sequence variations between the two assemblies.

Variation_type	Count	Length_hap1	Length_hap2
Syntenic regions	1946	930429859	933220504
Inversions	214	173602170	179045073
Translocations	2810	25628118	25466995
Duplications (hap1)	606	5530902	—
Duplications (hap2)	2237	—	9723891
Not aligned (hap1)	5069	300485152	—
Not aligned (hap2)	6795	—	274358289
SNPs	9487343	9487343	9487343
Insertions	337977	—	5279269
Deletions	347207	—	5438063
Copygains	244	—	3435634
Copylosses	242	—	1848175
Highly diverged	64150	706404286	713036945

## Data Records

The whole genome sequence data reported in this paper have been deposited in the Genome Warehouse^44^ in National Genomics Data Center, Beijing Institute of Genomics, Chinese Academy of Sciences/China National Center for Bioinformation, under accession number GWHEUSQ00000000.1 and GWHEUSR00000000.1 that is publicly accessible at https://ngdc.cncb.ac.cn/gwh. The raw sequence data reported in this paper have been deposited in the Genome Sequence Archive^45^ in National Genomics Data Center (Nucleic Acids Res 2022), China National Center for Bioinformation/Beijing Institute of Genomics, Chinese Academy of Sciences (GSA: CRA017679) under the BioProject accession number PRJCA026852 that are publicly accessible at https://ngdc.cncb.ac.cn/gsa, including Pacbio HiFi sequencing data (CRX1135011), ONT ultra-long sequencing data (CRX1135012), Hi-C sequencing data (SRR25859129), Illumina mixed-samples RNA sequencing data (CRR1232453), ONT mixed-samples RNA sequencing data (CRR1232454).

The data mentioned above has also been submitted to the National Center for Biotechnology Information (NCBI) SRA database. The data can be accessed under the bioproject PRJNA1192404, with the accession numbers SRR31557923 to SRR31557927^46–50^. Furthermore, the genome assembly results have been deposited in the DDBJ/ENA/GenBank, with accession numbers for the two haploid genomes designated as JBJNHD000000000^51^ and JBJNHE000000000^52^, respectively. Additionally, genome repeat annotations and functional annotations—including files for coding sequences (CDS), protein-coding regions, repeat annotations, and documentation of genetic variations between the two haploids—have been submitted to Figshare^53^.

## Technical Validation

### Evaluation of the sequenced read and quality control

Firstly, the raw data obtained from sequencing was subjected to data filtering to remove adapters and low-quality reads, yielding high-quality clean data. For the PacBio Circular Consensus Sequencing (CCS) data, we employed the ccs tool^54^. In the case of Nanopore Ultra-Long DNA Sequencing, we utilized Porechop (V0.2.4) for data processing^55^. For second-generation sequencing files, Trimmomatic was applied for quality trimming^56^. Subsequently, an assessment of data contamination in the clean data was conducted. The presence of contamination in the data can affect the accuracy of subsequent analyses, leading to significant deviations in genome features evaluations such as genome size, heterozygosity rate, and ultimately impacting the effectiveness of subsequent genome assembly. Therefore, 2,000 reads were randomly selected from the clean data and aligned against the NT database to assess contamination. According to the evaluation criteria, if more than 1% of reads align to evolutionarily distant species, it indicates potential contamination. Following the assessment, only approximately 0.3% of the data could be aligned to *Chenopodium quinoa*, demonstrating the absence of significant contamination. Finally, using visNano v0.1.1 (https://github.com/renzilin/visNano), the base quality was evaluated, demonstrating the exceptionally high quality of the original sequencing data (Fig. 1). Overall, the sequencing data was deemed to be of high purity and quality.

### Evaluation of the assembled genome

**Fig. 5:**
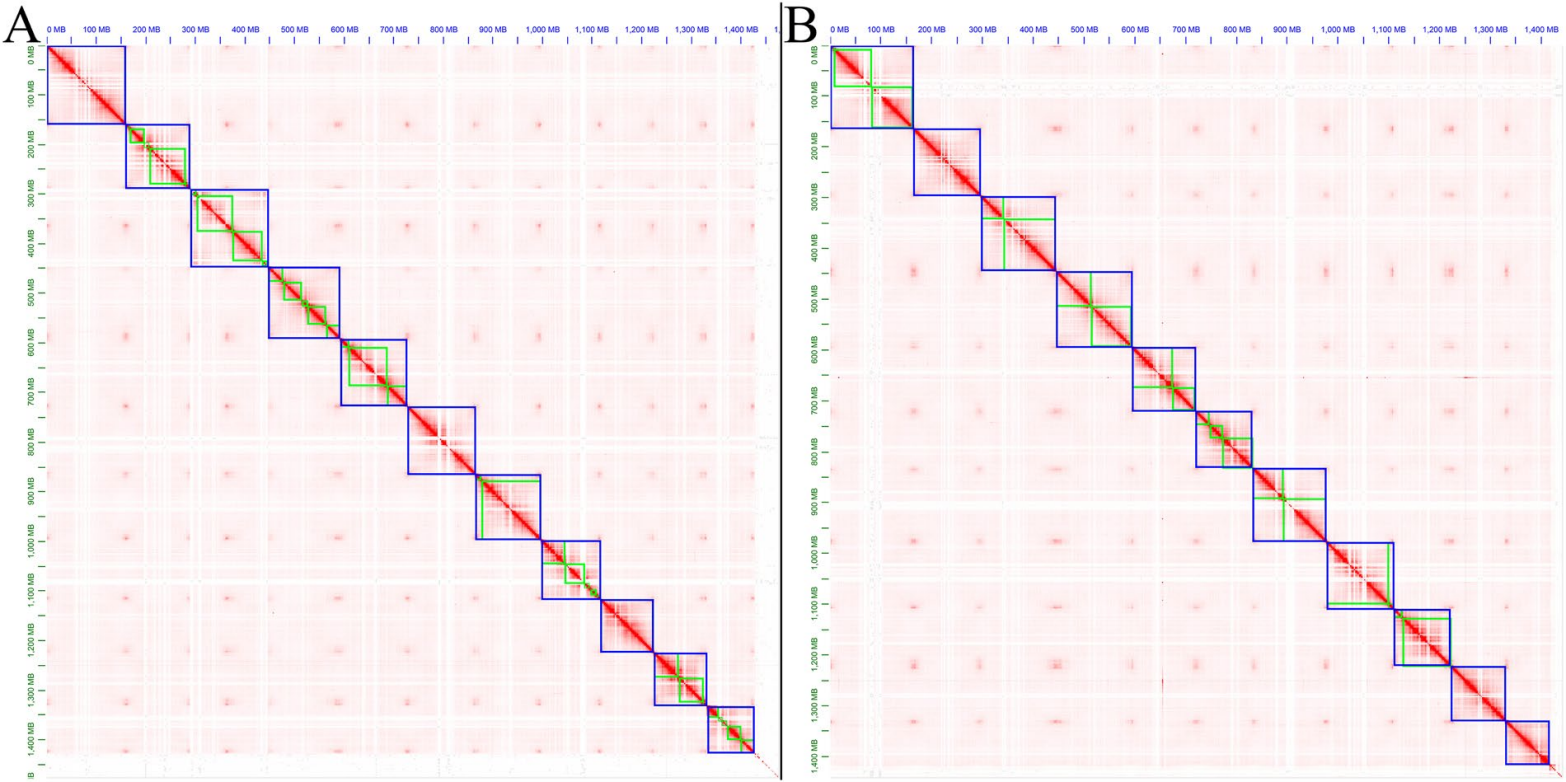
The Hi-C heatmap of chromosome interactions in *Selenicereus polyrhizus* chromosomes, panels A and B corresponding to haplotype 1 and haplotype 2, respectively.

We employed multiple methods to assess the accuracy and completeness of the assembly results. Firstly, based on high-quality ONT Ultra-long reads, PacBio HiFi reads, and Hi-C-assisted assembly, the preliminary assembly was scaffolded using YaHS^9^ (Yet Another Hi-C Scaffolding tool) v1.2a.1, generating a chromatin contact matrix. Visualization was performed using Juicebox^10^ Assembly Tools v2.20.00, revealing that both haplotypes of the assembly exhibited good contiguity, distinctly resolving 11 chromosomes (Fig. 5).

Secondly, TeloExplorer from quarTeT^12^ v1.2.1 was utilized to identify telomeres in the two haplotypes of the assembly, revealing the presence of 42 telomeres. Interestingly, both haplotypes of the assembly exhibited a lack of one telomere on chromosome 11 (Fig. 3).

Thirdly, the completeness of the genome was assessed using BUSCO^33^ v5.3.2 (genome mode, default parameters) with the embryophyta_odb10 dataset, validating that the two haplotypes achieved scores of 97.7% and 97.4%, respectively, including single copy and duplicated BUSCOs.

Lastly, merqury^57^ v1.3 was utilized to evaluate the consensus and completeness of the two haplotypes’ assembly using PacBio HiFi reads, following the recommended merqury algorithm with K-mer = 20. The quality values (QV) for haplotype 1 and haplotype 2 were determined to be 54.5 and 56.0, respectively. The completeness of the two haplotypes and the combined set was found to be 72.19%, 69.77%, and 96.58%, respectively. The aforementioned analyses collectively provide evidence of the accuracy and completeness of the genome assembly.

### Evaluation of the gene annotation

The two haplotypes were annotated with 29,139 and 28,538 protein-coding genes, respectively. Subsequently, the completeness of the annotated proteins was evaluated using BUSCO^33^ v5.3.2 (protein mode, default parameters). Notably, both haplotypes achieved completeness scores of 97.6% and 97.5%, respectively, indicating high quality of the gene annotation.

## Data Availability

No specific script was used in this work. The codes and pipelines used in data processing were all executed according to the manual and protocols of the corresponding bioinformatics software. The specific versions of software have been described in Methods.
